# Cross-Cultural Adaptation and Validation of the Methotrexate Intolerance Severity Score Questionnaire in Portuguese (Brazil) for Children and Adolescents with Juvenile Idiopathic Arthritis

**DOI:** 10.3390/jcm12031116

**Published:** 2023-01-31

**Authors:** Ana Carolina Londe, Jaqueline Cristina de Amorim, Paulo Rogério Julio, Nico M. Wulffraat, Roberto Marini, Simone Appenzeller

**Affiliations:** 1 Physiopathology Program, School of Medical Science, University of Campinas, Campinas 13083-970, Brazil; 2 Child and Adolescent Health Program, School of Medical Science, University of Campinas, Campinas 13083-970, Brazil; 3 Department of Pediatric Rheumatology, Wilhelmina Children’s Hospital, University Medical Center Utrecht, 3584 EA Utrecht, The Netherlands; 4 Department of Pediatrics, School of Medical Science, University of Campinas, Campinas 13083-970, Brazil; 5 Department of Orthopedics, Rheumatology and Traumatology, School of Medical Science, University of Campinas, Campinas 13083-970, Brazil

**Keywords:** juvenile idiopathic arthritis, methotrexate, intolerance, questionnaire validation

## Abstract

The Methotrexate (MTX) Intolerance Severity Score (MISS) questionnaire has been developed to identify MTX adverse events in juvenile idiopathic arthritis (JIA). The objective of this study was to translate and validate MISS into Brazilian Portuguese for children and adolescents. The MISS was translated into Portuguese following the standardized guidelines. We analyzed the following psychometric properties: acceptability, internal consistency, test–retest reproducibility, relative–child reliability, and external criterion and discriminant validity. We included 138 JIA patients (age: 8–18 years) and 108 relatives who took less than 5 min to answer MISS. Reproducibility tested after 15 days was good, with a kappa > 0.76. We observed good internal consistency (Cronbach’s coefficient 0.75–0.87 (patients) and 0.75–0.79 (relatives)). Reliability between patients and relatives was good except for stomachache and restlessness. Cut-off points of 5 and 6 had good sensitivity (84 and 71, respectively) and specificity (80 and 87, respectively). Using a cut-off value of 6, we observed 86 (62.3%) MTX-intolerant patients. In conclusion, MISS is a viable and practical tool for routine clinical care to identify MTX intolerance in JIA. Parents do not easily identify stomachache and restlessness as adverse MTX events.

## 1. Introduction

Juvenile idiopathic arthritis (JIA) is a clinically heterogeneous chronic inflammatory disease in patients with disease onset before 16 years of age [1]. Methotrexate (MTX) was synthesized in the 1940s as an antineoplastic drug, and was used as a therapy for arthritis and psoriasis in 1951 [2,3]. For several JIA subtypes, MTX is considered to be the first choice of disease-modifying antirheumatic drug (DMARD). At standard doses (10–15 mg/m^2^/week), 60–75% of JIA patients have significant improvement [4]. MTX can be used as monotherapy or in combination with other synthetic or biologic DMARDs [5,6]. Serious adverse effects are rarely observed with MTX use and usually remit with the interruption of the drug. However, gastrointestinal events, such as nausea, vomiting, and/or abdominal pain, are frequently reported [7]. In addition, anticipatory symptoms associated with MTX treatment were reported in JIA [7]. In order to evaluate the entire spectrum of MTX intolerance, Bulatovic et al., developed and validated a questionnaire called the MTX Intolerance Severity Scale (MISS) that included 4 domains: stomachache, nausea, vomiting, and behavioral complaints [8]. The MISS has been used in a number of studies with JIA and RA patients, and has been validated in several languages [9,10,11,12,13,14,15,16]. In Brazil, a validation process exists for adults with rheumatoid arthritis (RA), but there isn no cross-cultural adaptation for children [17]. Therefore, the aim of this study was to cross-culturally adapt MISS for children with JIA into Brazilian Portuguese. In addition, we determined factors associated with MTX intolerance.

## 2. Materials and Methods

We invited consecutive JIA patients and their relatives to participate in the study during their regular visit at the pediatric rheumatology unit at the Clinics Hospital of the University of Campinas between August 2017 and June 2019. To be included, children and adolescents had to be between 8 and 18 years of age, fulfill ILAR criteria for JIA [18], and have received an MTX treatment (dose between 5 and 15 mg/m^2^/week) for more than 3 months independent of the route of administration. It was a convenience sample, and we aimed to include a minimum of 10 JIA patients per MISS questionnaire item [19,20].

In total, 138 patients and 108 relatives filled out the questionnaires in different rooms, and we measured the time spent to answer it. A total of 36 patients repeated the questionnaire after 15 days to test the reproducibility.

Demographic and disease-related characteristics were collected through medical chart review.

The study was approved by the local IRB (CAAE = 69672717.5.0000.5404), and informed written consent was obtained from each subject and/or legal guardian.

### 2.1. Translation and Transcultural Adaptation

Prior authorization was obtained from the authors of the original questionnaire (NW) [8]. The MISS was translated into Portuguese (Brazil) following standardized guidelines [21,22]. Two independent bilingual translators (one familiar with the medical terminology and context of the questionnaire, the other with no medical background) translated the questionnaire into Portuguese (Brazil). They produced two independent translations (T1 and T2). Posteriorly, a synthesis of these translations was produced (T12). Version T12 was then back translated by two different translators fluent in English (one familiar with the medical terminology and context of the questionnaire, the other with no medical background) (BT1 and BT2). The synthesis of this translation was approved by one of the original participants of the study (NW). An expert committee of 10 native Portuguese speakers (pediatric rheumatologists and family members of patients) defined the prefinal version of the questionnaire. They analyzed cross-cultural equivalence, so we worked on semantic, idiomatic, experimental, and conceptual aspects of the questionnaire. For the cognitive debriefing: 10 native Portuguese speakers (physicians, patients, and general population) were asked to determine the clarity of each item of the final questionnaire in Portuguese. Lastly, a pilot test was applied to 36 children with JIA and 20 relatives. The first test was followed by a retest after a 15-day interval.

### 2.2. Statistical Analysis

Data analysis was performed on SPSS^®^ software, version 21, and R, version 4.0.3. The sample size was calculated on the basis of the formula by Kothari [23]. Considering our target population of 150, estimated variance of 0.5 (50% of MTX intolerance), confidence level 95% (z = 1.96), desired level of precision 0.03, and response rate of 90% (based on a previous result), we obtained a minimal sample size of 125. We used the COSMIN reporting standards to describe the psychometric results [23]. The following psychometric properties were evaluated and are reported: acceptability, internal consistency (Cronbach’s alpha coefficient), test–retest reproducibility, relative–child reliability, external validity, and criterion validity [24,25,26]. Factor analysis was used for convergent and discriminant validity.

We plotted the ROC curve to evaluate the discriminant validity of the translated MISS questionnaire compared to the gold standard, which is based on clinical interviews and symptoms of adverse events of MTX recorded in medical charts. The cut-off score for intolerance was determined by analyzing sensitivity and specificity.

Principal component analysis (PCA) was conducted to determine variability in the principal components.

## 3. Results

### 3.1. Descriptive Data

We included 138 subjects with JIA (101 (73.2%) women) with a median age of 12.3 years (range, 8–18 years). Parenteral MTX was used by 111 individuals (80.4%). The median time of MTX use was 4.87 years (range, 3 months–6.2 years)

### 3.2. Cross-Cultural Adaptation

We found minor difficulties in translate the MISS, especially in the description of “several hours to 1 day before taking MTX”. Minor transcultural adaptation was needed regarding the description of the frequency of intolerance symptoms in Portuguese (Brazil). The original version of “no, mild, moderate, and severe symptoms” was changed into “never, sometimes, often and always”. These words facilitated the understanding of the scores by our target population.

### 3.3. Psychometric Issues

Psychometric characteristics of the translated version are reported below:

**Acceptability:** patients and relatives required less than 5 min to respond to the questionnaire.

**Internal consistency:** The translated questionnaire had a Cronbach’s alpha of 0.88. Individual items had Cronbach’s alpha values ranging from 0.75 to 0.87 for JIA patient responses (Table 1), and from 0.75 to 0.79 in the responses of the relatives. No significant difference was observed when excluding one item of the questionnaire.

Regarding interitem correlation, values are shown in Table 2. The convergent validity was good (KMO = 0.885). Using factor analysis, we observed that the construct was divided into 3 components. Component 1 had an average factor analysis of 0.65, Component 2 of 0.73, and Component 3 of 0.68. Discriminant validity was good, with the component variance being greater than the square of correlations in each component (variance of extracted Component 1 = 0.45, square correlation = 0.22; variance of extracted Component 2 = 0.55, square correlation = 0.15; variance of extracted Component 3 = 0.49, square correlation = 0.16).

**Test–retest reproducibility:** Reproducibility was assessed in 36 JIA patients. The concordance to both test and retest was very good, in the range of 0.76–1. In both test and retest, patients used the same route of MTX administration (Table 3).

**Relative–child reliability**: The concordance between relatives and children was variable and ranged from 0.45 to 0.96 (Table 4). The lowest concordance was observed in Items 9 (I feel restless when taking MTX), 2 (I have a stomachache a day before taking MTX), and 11 (I feel irritable when taking MTX).

**External validity:** we observed acceptable external validity with the correlation between MISS, and clinical interviews and chart reviews (correlation coefficient r = 0.74).

**Figure 1:**
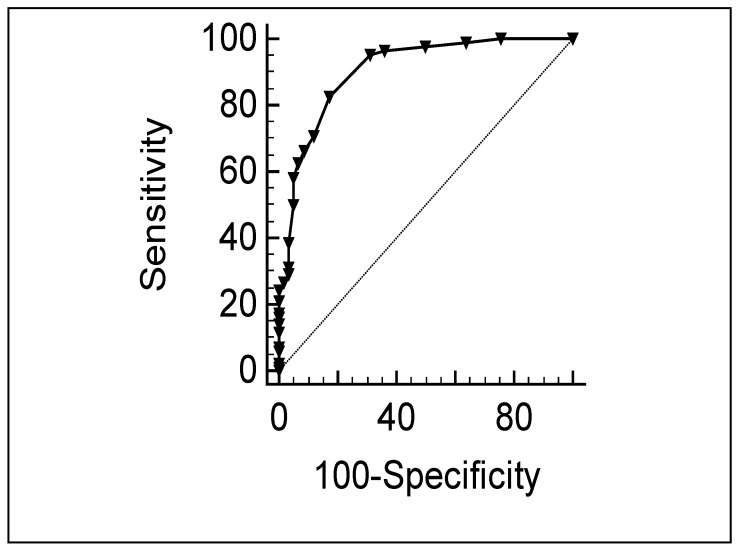
ROC curve plotting MTX intolerance with the questionnaire against the gold standard with cut-off scores of 5, including JIA and relative responses. AUC = 0.90 (95% CI, 0.85–0.94).

**Figure 2:**
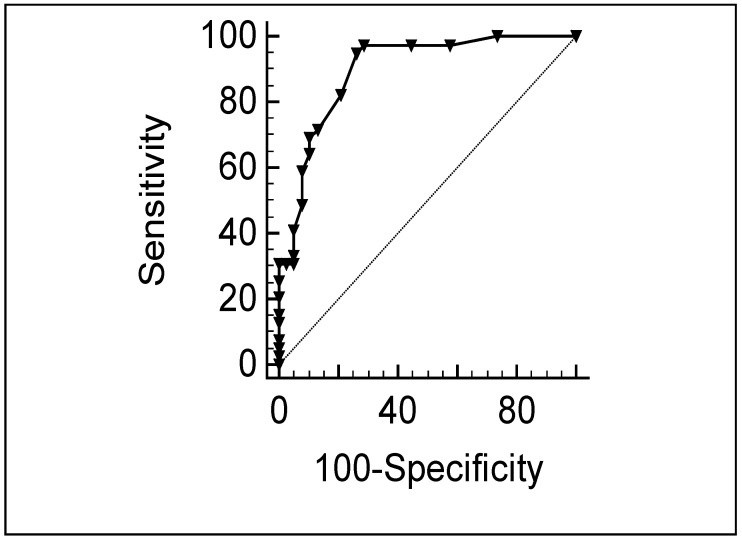
ROC curve plotting MTX intolerance with the questionnaire against the gold standard with cut-off score of 5 in JIA patients. AUC = 0.89 (95% CI, 0.80–0.95).

**Criterion validity:** We plotted the ROC curve, and the area under the curve (AUC) was 0.90 (95% CI, 0.85–0.94) for the total sample (children and relatives) (Figure 1) and 0.89 (95% CI, 0.80–0.95) (Figure 2) for JIA patients. Sensitivity and specificity for the intolerance cut-off scores are shown in Table 5. We observed good concordance when using cut-off scores of 5 (kappa, 0.73) and 6 (kappa, 0.79).

**Figure 3:**
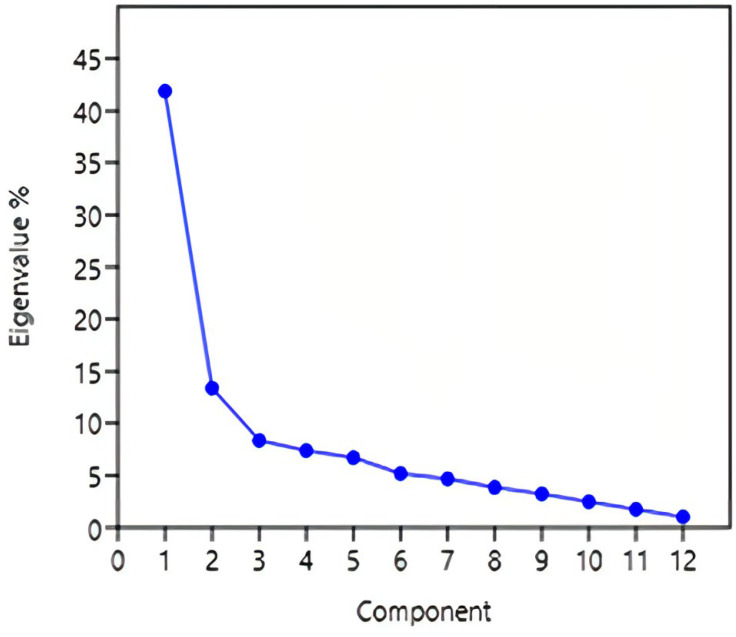
Principal component analysis of the translated MISS score.

**Principal component analysis:** the two first components were responsible for 54% of the observed variability, as shown in Figure 3.

**Figure 4:**
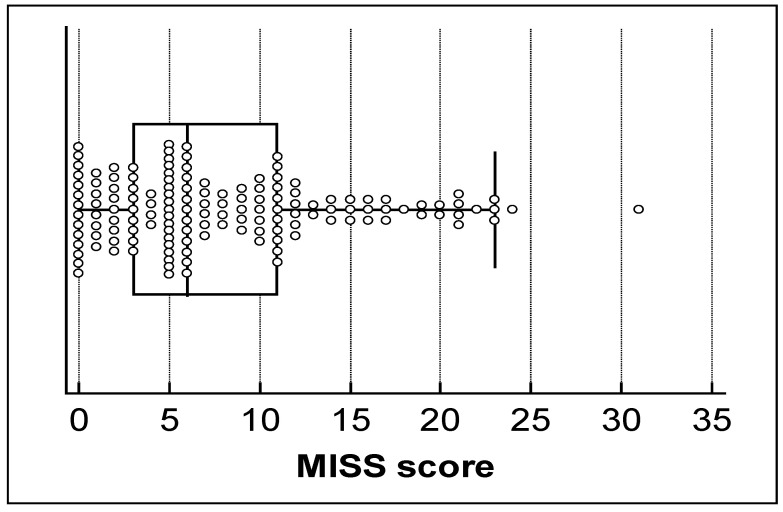
Distribution of total MISS scores in JIA patients. The MISS score was from 0 to 36 points.

**Variables associated with MTX intolerance:** The median score for the MISS in our JIA cohort was 6.0 points (Figure 4). We observed that 14 (10%) patients had a score of 0, and no patient had the maximal score. Therefore, we excluded the floor and ceiling effects of the validated MISS questionnaire (score < 15%). We identified 86 (62.3%) intolerant patients and 22 (37.6%) tolerant patients with a cut-off of 6 points. In intolerant patients, the median score of the MISS was 12.82 points, whereas tolerant patients had a median value of 2.42 points. Intolerance was more frequently observed in patients taking subcutaneous MTX (*p* = 0.03). No association with age (*p* = 0.35) and MTX treatment duration (*p* = 0.54) was observed.

## 4. Discussion

The MISS was used in both JIA and RA patients using MTX [9,10,11,12,13,14,15,16,17]. A French translation study used patients with JIA and their relatives, but the Brazilian study included only adult patients with RA, and no test–retest were performed [16,17]. Our study translated and adapted the MISS into Brazilian Portuguese for patients with JIA and their respective relatives; then, we obtained the psychometric properties according to standardized mathods [21]. The psychometric values of the Portuguese (Brazil) MISS for children and adolescents were similar to those in previous translations [16,17]. Internal consistency was acceptable to good. Reproducibility was good to very good. Reliability in children–relatives was variable, with lower scores in items related to stomachache (κ = 0.50–0.60) and restlessness when taking MTX (κ = 0.45). The French version also found lower reliability in children–relatives regarding anticipatory stomachache (κ = 0.33) and restlessness (κ = 0.40) in child–parent pairs, indicating the difficulty in parents identifying these important side effects related to MTX [16].

A cut-off score of 5 or 6 for children and relatives yielded the best sensitivity and specificity to discriminate between MTX intolerance and tolerance, similar to the original version [8]. The French version found a cut-off of 3 to discriminate tolerant from intolerant JIA patients [16]. Regarding cut-off MISS scores of 5 and 6, we found good concordance between the two completions, with kappas of 0.73 and 0.79, respectively. Other studies that used the MISS as tool to differentiate between tolerance and intolerance to MTX in JIA and RA, with intolerance having more than 6 points [8,9,10,11,12,13,14,21]. Since the adult version in Portuguese (Brazil) determined the best cut-off to be 6 points, we considered 6 to be the most adequate cut-off point to provide an adequate transition of children with JIA into adulthood [17].

We observed that approximately 62% of our JIA patients had a degree of MTX intolerance. In the literature, MTX intolerance in JIA varies in the range of 40–70% [27,28]. The median score of MISS-intolerant patients was similar to that in the original version [8]. We observed a greater intolerance in JIA patients taking subcutaneous MTX than what had previously been reported [8,26,27]. No association with age or disease duration was observed in our study.

This study has some limitations. It was a single-center, cross-sectional study, and a convenience sample was used for validation. This validation was performed during the same period of the validation of the adult RA MISS scores [17]. The text had some differences, since our target population was children older than 8 years, but the psychometric properties were similar, and the instrument could be used for transition. External validation in different Brazilian cohorts is of interest to replicate the results.

Longitudinal studies are of interest to determine clinical and psychological factors associated with MTX intolerance, and strategies to reduce its occurrence and increase adherence.

## 5. Conclusions

In conclusion, the MISS can be used in clinical practice to determine the prevalence of MTX intolerance in JIA patients and improve patient adherence.

## Figures and Tables

**Table 1:** Internal consistency of the Portuguese version of MISS.

Item	Total Item Correlation	Cronbach’s Alpha
1	0.62	0.87
2	0.46	0.78
3	0.64	0.76
4	0.72	0.75
5	0.67	0.76
6	0.68	0.75
7	0.61	0.76
8	0.47	0.77
9	0.66	0.75
10	0.56	0.88
11	0.69	0.87
12	0.76	0.87

MISS questionnaire—12 items: 1 = I have a stomachache after taking MTX; 2 = I have a stomachache a day before taking MTX; 3 = I have stomachache when thinking MTX; 4 = I am nauseous after taking MTX; 5 = I am nauseous a day before taking MTX; 6 = I am nauseous when thinking of MTX; 7 = I vomit after taking MTX; 8 = I vomit a day before taking MTX; 9 = I feel restless when taking MTX; 10 = I cry when taking MTX; 11 = I feel irritable when taking MTX; 12 = I refuse to take MTX [8].

**Table 2:** Convergent validity (interitem Pearson’s r correlations) of the Portuguese version of MISS.

Items												
	**1**	2	3	4	5	6	7	8	9	10	11	12
**1**	*1*											
**2**	0.40 **	*1*										
**3**	0.54 **	*0.45*	*1*									
**4**	0.56 **	*0.32*	*0.41*	*1*								
5	0.39 **	*0.42*	*0.40*	*0.56*	*1*							
6	0.42 **	*0.40*	*0.43*	*0.64*	*0.55*	*1*						
7	0.38 **	*0.31*	*0.34*	*0.51*	*0.50*	*0.38*	*1*					
8	0.24 **	*0.28*	*0.20*	*0.18*	*0.39*	*0.26*	*0.43*	*1*				
9	0.37 **	*0.22*	*0.34*	*0.42*	*0.37*	*0.39*	*0.32*	*0.32*	*1*			
10	0.16 *	*0.16*	*0.29*	*0.26*	*0.32*	*0.28*	*0.41*	*0.50*	*0.43*	*1*		
11	0.30 **	*0.23*	*0.38*	*0.43*	*0.40*	*0.43*	*0.30*	*0.34*	*0.69*	*0.57*	*1*	
12	0.46 **	*0.27*	*0.43*	*0.51*	*0.49*	*0.50*	*0.42*	*0.31*	*0.60*	*0.49*	*0.65*	*1*

MISS questionnaire—12 items: 1 = I have a stomachache after taking MTX; 2 = I have a stomachache a day before taking MTX; 3 = I have stomachache when thinking MTX; 4 = I am nauseous after taking MTX; 5 = I am nauseous a day before taking MTX; 6 = I am nauseous when thinking of MTX; 7 = I vomit after taking MTX; 8 = I vomit a day before taking MTX; 9 = I feel restless when taking MTX; 10 = I cry when taking MTX; 11 = I feel irritable when taking MTX; 12 = I refuse to take MTX [8]. * *p* < 0.05; ** *p* < 0.001.

**Table 3:** Child test and retest results of the Portuguese version of MISS.

Item	Kappa
1	0.87
2	*0.81*
3	0.76
4	0.88
5	0.86
6	0.80
7	0.95
8	1.0
9	*1.0*
10	0.92
11	*0.81*
12	0.84

MISS questionnaire—12 items: 1 = I have a stomachache after taking MTX; 2 = I have a stomachache a day before taking MTX; 3 = I have stomachache when thinking MTX; 4 = I am nauseous after taking MTX; 5 = I am nauseous a day before taking MTX; 6 = I am nauseous when thinking of MTX; 7 = I vomit after taking MTX; 8 = I vomit a day before taking MTX; 9 = I feel restless when taking MTX; 10 = I cry when taking MTX; 11 = I feel irritable when taking MTX; 12 = I refuse to take MTX [8].

**Table 4:** Child–relative reliability of the Portuguese version of MISS.

Item	Kappa	Agreement Percentage (%)
1	0.66	82
2	*0.50*	79
3	0.66	84
4	0.83	91
5	0.86	90
6	0.70	96
7	0.94	95
8	0.81	94
9	*0.46*	89
10	0.90	90
11	*0.50*	89
12	0.80	95

MISS questionnaire—12 items: 1 = I have a stomachache after taking MTX; 2 = I have a stomachache a day before taking MTX; 3 = I have stomachache when thinking MTX; 4 = I am nauseous after taking MTX; 5 = I am nauseous a day before taking MTX; 6 = I am nauseous when thinking of MTX; 7 = I vomit after taking MTX; 8 = I vomit a day before taking MTX; 9 = I feel restless when taking MTX; 10 = I cry when taking MTX; 11 = I feel irritable when taking MTX; 12 = I refuse to take MTX [8].

**Table 5:** Sensitivity and specificity for cut-off scores (2–11 points) on the MISS.

Cut-Off Scores	Sensitivity	Specificity
2	98	50
3	96	64
4	95	68
5	84	80
6	71	87
7	66	91
8	63	93
9	58	95
10	50	96
11	40	98

## Data Availability

The raw data supporting the conclusions of this article will be made available by the authors without undue reservation.
